# A 10-pathway burden score complements tumor mutational burden for outcome stratification in non-small cell lung cancer

**DOI:** 10.3389/fmed.2026.1882870

**Published:** 2026-08-06

**Authors:** Feng Mao, Ning Wang, Yuming Wang, Ziang Wang, Hui Zhang, Qingquan Luo

**Affiliations:** 1 Department of Thoracic Surgery, Shanghai Chest Hospital, Shanghai Jiao Tong University, Shanghai, China; 2 Department of Oncology, Shanghai Chest Hospital, School of Medicine, Shanghai Jiao Tong University, Shanghai, China

**Keywords:** 10-pathway burden, non-small cell lung cancer, notch pathway, oncogenic pathways, treatment response, tumor mutational burden

## Abstract

**Background:**

Tumor mutational burden (TMB) summarizes the number of nonsilent mutations in a tumor but does not show whether these alterations cluster within a small set of pathways or extend across several oncogenic programs. To address this distinction, we developed a 10-pathway burden (10-PB) framework and defined the 10-PB score as the number of altered canonical oncogenic pathways per tumor. We then evaluated whether this mutation-based measure of pathway breadth provides information complementary to TMB in non-small cell lung cancer (NSCLC).

**Methods:**

Nonsilent mutations were mapped to 10 canonical oncogenic pathways to construct the 10-PB score. We first described pathway-level alteration patterns and then examined associations of 10-PB with survival and treatment response. Its incremental value relative to TMB was assessed using multivariable models, likelihood-ratio tests, and Akaike information criterion. Bootstrap resampling, continuous-risk analyses, and leave-one-pathway-out models were used to evaluate the stability and endpoint-specific structure of the findings. External MSK-based cohorts were used for validation.

**Results:**

Most tumors showed alterations in 2–4 pathways, indicating that multi-pathway involvement is common in NSCLC. LUAD and LUSC showed distinct pathway-level profiles. Higher 10-PB was associated with shorter overall survival and progression-free survival after adjustment for clinicopathologic factors and TMB. Model comparisons suggested that 10-PB adds prognostic information to TMB-based models. In the treatment-response analysis, higher 10-PB showed an exploratory association with unfavorable response. Leave-one-pathway-out analyses suggested endpoint-specific patterns, with NOTCH more closely associated with progression-free survival and adverse response, and TGF-β and PI3K more closely associated with overall survival. External cohorts showed broadly consistent results.

**Conclusion:**

The 10-PB framework extends mutation-based assessment of NSCLC by describing how broadly nonsilent mutations involve canonical oncogenic pathways. This pathway-breadth measure complements TMB and may help refine prognosis-oriented stratification, while its treatment-response associations should be viewed as exploratory. Prospective validation and integration with functional pathway-activity data are needed before clinical application.

## 1 Introduction

Non-small cell lung cancer (NSCLC) remains a major clinical challenge worldwide (1). Lung adenocarcinoma (LUAD) and lung squamous cell carcinoma (LUSC), the two major histologic subtypes, account for most NSCLC cases and differ substantially in molecular features and clinical behavior (2). Despite advances in molecular diagnostics, targeted therapy, immunotherapy, and multimodal treatment, many patients still experience poor outcomes, and intertumoral diversity continues to complicate prognostic stratification and treatment selection (3–5). A pathway-level view may help place somatic alterations into a more clinically interpretable context.

Large-scale genomic studies have shown that LUAD and LUSC differ not only in histology but also in recurrent somatic alterations (2, 6, 7). LUAD is commonly characterized by alterations in genes such as EGFR, KRAS, STK11, and KEAP1, whereas LUSC more often harbors alterations involving TP53, PIK3CA, CDKN2A, NFE2L2, and related genes (6, 8–11). These gene-centered studies have substantially advanced the molecular classification of NSCLC. At the same time, the long-tail distribution of mutations and the recurrence of complex co-alteration patterns make it difficult to interpret tumor heterogeneity from individual genes alone (12). TMB offers a useful summary of mutation load, but tumors with similar TMB may differ in whether their mutations affect one dominant pathway or several oncogenic programs. This difference in pathway breadth is not captured by mutation counts alone.

Pathway-based analysis provides an intermediate level of interpretation between individual genes and global mutation burden. The PanCancer Atlas framework described by Sanchez-Vega et al. (9) classifies recurrent cancer-associated alterations into 10 canonical oncogenic pathways, including RTK/RAS, PI3K, Cell cycle, TP53, NRF2, WNT, NOTCH, TGF-β, MYC, and Hippo (13). This framework summarizes diverse genomic events into functionally related signaling programs and allows pathway-specific frequencies, co-alteration patterns, and cumulative pathway involvement to be examined (14). In NSCLC, where alterations often span more than one oncogenic program, a mutation-based measure of pathway breadth may add interpretability when considered alongside TMB and mutated-gene count.

In this study, we developed a 10-pathway burden framework to quantify mutation-based pathway-level complexity in NSCLC. Nonsilent mutations were mapped to canonical oncogenic pathways, and the resulting 10-PB score was defined as the number of altered pathways per tumor. We characterized pathway alteration patterns in LUAD and LUSC, evaluated the associations of 10-PB with survival and treatment response, and tested whether 10-PB provides information complementary to TMB. We also assessed robustness, examined endpoint-specific model-based pathway associations, and validated selected findings in independent NSCLC cohorts.

## 2 Materials and methods

### 2.1 Study cohorts and clinical endpoints

This study used the TCGA NSCLC cohort as the discovery cohort. Tumors with available somatic mutation profiles and clinicopathologic annotations were included. Lung adenocarcinoma (LUAD) and lung squamous cell carcinoma (LUSC) were analyzed as the two major histologic subtypes. Clinical variables included histology, sex, age, TNM stage, survival outcomes, and treatment response. Age was grouped as <60 or ≥60 years for descriptive analyses and modeled per 10-years increase in regression models. T stage was grouped as T1–2 or T3–4, N stage as N0 or N+, and M stage as M0 or M1. Overall stage was used in survival models, with stage I as the reference category. Overall survival (OS) and progression-free survival (PFS) were defined according to TCGA endpoint annotations. Treatment response was classified as favorable response, including complete response and partial response (CR/PR), or adverse response, including stable disease and progressive disease (SD/PD). Because response annotations may reflect heterogeneous treatment settings, treatment-response analyses were considered exploratory. External validation was performed using four independent MSK-based cohorts: MSKCC 2020 and LUAD-MSKCC 2023 Met for OS validation, LUAD-MSKCC 2020 for recurrence-free survival validation, and NSCLC PD1 MSK 2018 for PD-1/PFS-related and NOTCH-associated analyses.

### 2.2 Somatic mutation processing and mutation-load comparators

Analyses were restricted to nonsilent somatic mutations, including missense, nonsense, frameshift, in-frame insertion/deletion, splice-site, translation-start-site, and nonstop mutations. Synonymous and noncoding variants were excluded. Tumor mutational burden (TMB) was defined as the total number of nonsilent mutation events per tumor and served as the primary mutation-count-based comparator for the 10-pathway burden framework. Mutated-gene count was defined as the number of genes carrying at least one nonsilent somatic mutation in each tumor and was examined as an additional mutation-load measure. For visualization, mutation burden was log-transformed as log10(nonsilent mutation count + 1).

### 2.3 Construction of the 10-pathway burden framework

Canonical oncogenic pathways were defined according to the PanCancer Atlas PathwayMapper framework, including RTK/RAS, PI3K, Cell cycle, TP53, NRF2, WNT, NOTCH, TGF-β, MYC, and Hippo. Gene symbols were harmonized using HGNC-approved nomenclature, and representative and full pathway gene lists are provided in Table 1, respectively. Nonsilent mutations were mapped to the corresponding pathway gene sets. A pathway was considered altered when at least one assigned gene carried a nonsilent mutation. Multiple mutations within the same pathway were counted once so that the score reflected pathway breadth rather than the number of mutations within a pathway.

**TABLE 1:** Canonical oncogenic pathways and representative genes included in this study.

Pathway	Representative genes
RTK/RAS	EGFR, KRAS, ERBB2, BRAF, MET, NF1
PI3K	PIK3CA, PTEN, AKT1, PIK3R1, TSC1, TSC2
Cell cycle	CDKN2A, CDK4, CCND1, RB1, CDKN2B, E2F3
TP53	TP53, MDM2, MDM4, ATM, CHEK2, TP63
NRF2	NFE2L2, KEAP1, CUL3
WNT	APC, CTNNB1, AXIN1, AXIN2, RNF43, FAT1
NOTCH	NOTCH1, NOTCH2, NOTCH3, NOTCH4, FBXW7, CREBBP, EP300, NCOR1
TGF-β	TGFBR1, TGFBR2, ACVR2A, SMAD2, SMAD3, SMAD4, BMPR2
MYC	MYC, MYCL, MYCN, MAX, MGA, MNT
Hippo	NF2, LATS1, LATS2, YAP1, WWTR1, SAV1

Representative genes are shown for clarity in the main text and do not represent the full harmonized pathway templates.

The 10-pathway burden framework refers to the overall analytical approach used to map nonsilent mutations to canonical oncogenic pathways. The 10-PB score refers to the numerical count of altered pathways per tumor, ranging from 0 to 10. For regression analyses, 10-PB and TMB were standardized and modeled per standard deviation increase. For Kaplan-Meier analyses, patients were grouped into low burden (0–1 altered pathways), intermediate burden (2–4 altered pathways), and high burden (≥5 altered pathways). These cutoffs were selected according to the observed distribution of 10-PB scores and their interpretability for distinguishing limited, intermediate, and broad pathway involvement. The distribution of 10-PB and its relationships with TMB and mutated-gene count were examined to distinguish pathway breadth from mutation load.

### 2.4 Pathway-level mutational architecture analyses

Pathway alteration frequency was calculated as the proportion of tumors with at least one nonsilent mutation in each pathway. Pairwise pathway associations were assessed using binary pathway alteration matrices. Co-altered pathway pairs were tested using Fisher’s exact test, and odds ratios were log2-transformed for visualization. False discovery rate (FDR) correction was applied using the Benjamini–Hochberg method.

Mutual exclusivity patterns were evaluated descriptively at the pathway or gene-pair level. Recurrent pathway modules were defined as combinations of altered pathways within each tumor and summarized by frequency. The most frequent module combinations were compared between LUAD and LUSC.

Subtype-specific pathway and gene composition analyses were also performed. For each canonical pathway, genes were ranked by subtype-specific mutation prevalence. Dominant altered genes were used to describe pathway composition in LUAD and LUSC and to provide gene-level context for subtype-biased pathway organization.

### 2.5 Clinicopathologic association analyses

Associations of mutation-load measures and 10-PB with clinicopathologic variables were evaluated in the TCGA cohort. Binary subgroup comparisons were performed using two-sided Wilcoxon rank-sum tests. Overall mutation burden was analyzed as log10(nonsilent mutation count + 1), whereas 10-PB was analyzed as the number of altered pathways.

### 2.6 Survival analyses

Associations of 10-PB with OS and PFS were evaluated using Cox proportional hazards regression. Univariable and multivariable models were fitted separately for OS and PFS. Covariates included histology, sex, overall stage, age, TMB, and 10-PB. Histology was modeled as LUSC versus LUAD. Overall stage was modeled using stage I as the reference category. Age was modeled per 10-years increase. TMB and 10-PB were modeled per SD increase. Hazard ratios (HRs) and 95% confidence intervals (CIs) were reported. Kaplan–Meier analyses were performed using predefined 10-PB groups, and survival differences were assessed using log-rank tests. TMB-based stratification was also evaluated to compare pathway-level burden with mutation-volume-based stratification.

### 2.7 Treatment-response analyses

The association between 10-PB and treatment response was assessed using logistic regression. SD/PD was defined as the adverse outcome, and CR/PR was used as the reference category. Univariable analyses were first performed for each clinicopathologic and genomic variable. A multivariable model was then fitted including histology, sex, overall stage, age, TMB, and 10-PB. TMB and 10-PB were modeled per SD increase. Odds ratios (ORs) and 95% CIs were reported.

### 2.8 Incremental value of 10-PB beyond TMB

To evaluate whether 10-PB added information to mutation-load-based models, nested model comparisons were performed for OS, PFS, and adverse treatment response. For each endpoint, a baseline model containing clinicopathologic covariates and TMB was compared with a model additionally including 10-PB. Conversely, a model containing clinicopathologic covariates and 10-PB was compared with a model additionally including TMB. Incremental model fit was assessed using likelihood-ratio tests and changes in Akaike information criterion (AIC).

### 2.9 Continuous-risk and robustness analyses

In addition to categorical grouping, 10-PB was examined as a continuous variable to assess whether increasing pathway burden showed a graded relationship with clinical risk. Continuous-risk plots were used to visualize the association between 10-PB and adjusted outcome risk for OS, PFS, and adverse treatment response. The reference value was set at 10-PB = 2 altered pathways. Bootstrap resampling was performed with 500 iterations by refitting the endpoint-specific multivariable models and resampling the 10-PB effect estimate. These analyses were used to support the categorical and regression-based findings rather than to define new endpoints.

### 2.10 Endpoint-specific pathway contribution analyses

Leave-one-pathway-out model comparisons were used to evaluate endpoint-specific model-based contributions of individual pathways to OS, PFS, and treatment response. For each endpoint, a full model including the 10 pathway indicators and relevant covariates was fitted. Reduced models were then generated by removing one pathway at a time. Each reduced model used the same covariates as the corresponding full model. The contribution of each pathway was quantified by likelihood-ratio testing, comparing the full model with the corresponding reduced model.

For selected pathways, Kaplan-Meier analyses were performed according to pathway alteration status. For treatment response, pathway alteration status was compared with the distribution of CR/PR and SD/PD. These analyses were interpreted as model-based statistical associations rather than evidence of direct biological causality.

### 2.11 External validation analyses

External validation applied the same pathway gene sets, nonsilent mutation definition, altered-pathway calling rule, and 10-PB calculation used in the discovery analysis. The reproducibility of the 10-PB framework was assessed by evaluating its association with clinical outcomes in independent cohorts. OS was evaluated in MSKCC 2020 and LUAD-MSKCC 2023 Met, and recurrence-free survival was evaluated in LUAD-MSKCC 2020. Survival associations were assessed using Kaplan–Meier and Cox regression analyses. When available, multivariable models were adjusted for clinicopathologic variables and TMB. Pathway-specific validation was performed for selected endpoint-related signals identified in the discovery cohort. PI3K and TGF-β alterations were evaluated for OS in LUAD-MSKCC 2023 Met. NOTCH alteration was evaluated for PFS and treatment-associated subgroup signals in NSCLC PD1 MSK 2018.

### 2.12 Statistical analysis

All analyses were performed in R. Unless otherwise specified, tests were two-sided, and *P* < 0.05 was considered statistically significant. FDR correction was applied using the Benjamini–Hochberg method for pairwise pathway-association analyses. Analyses not explicitly described as FDR-adjusted were considered exploratory.

## 3 Results

### 3.1 Development of the 10-PB framework reveals structured pathway-level mutational patterns

**FIGURE 1:**
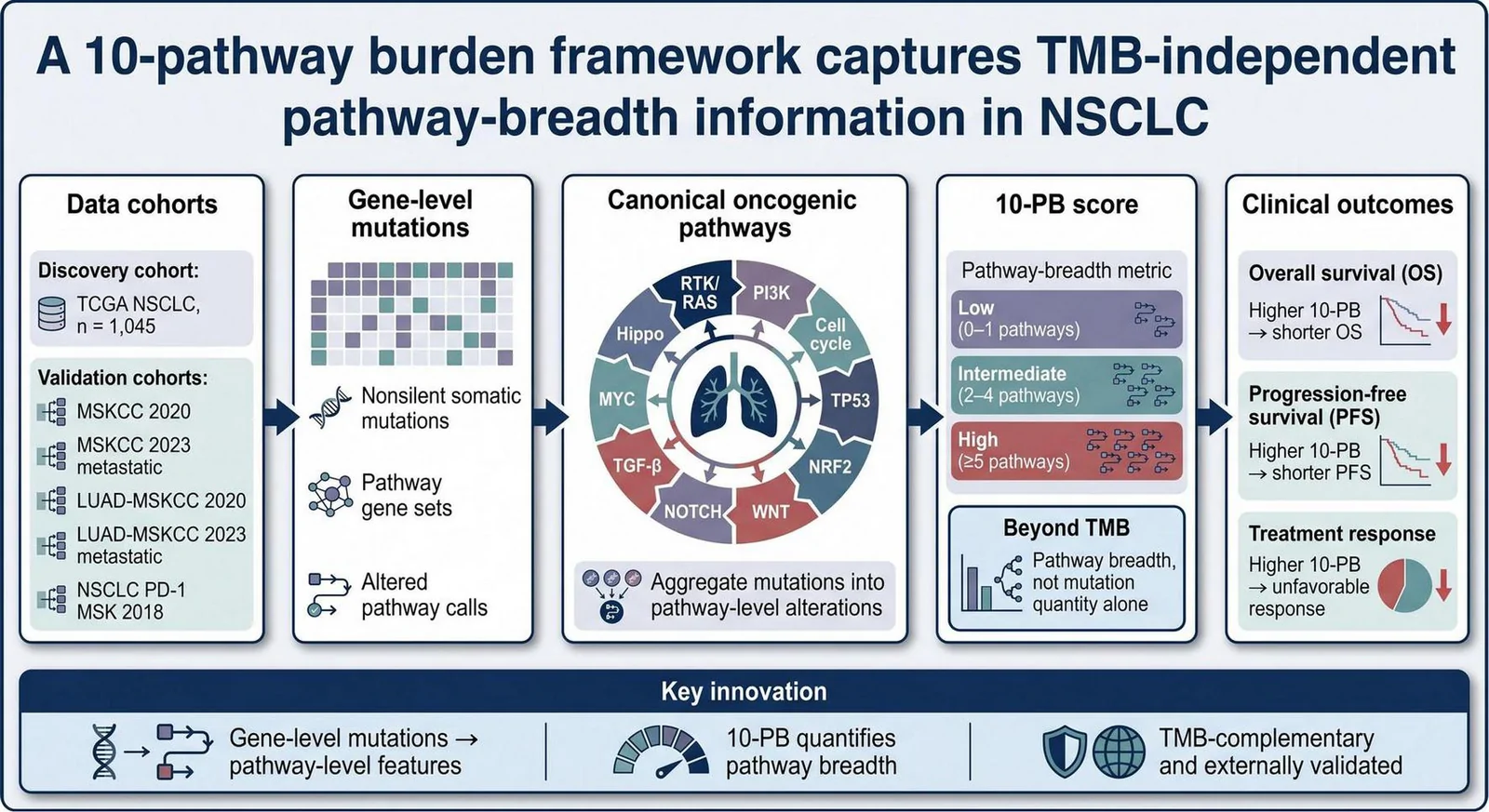
Study design and analytical workflow. Somatic mutations from the TCGA NSCLC discovery cohort were mapped to 10 canonical oncogenic pathways to construct the 10-pathway burden (10-PB) score. The workflow included survival, treatment-response, model-comparison, robustness, pathway-specific, and external validation analyses.

**FIGURE 2:**
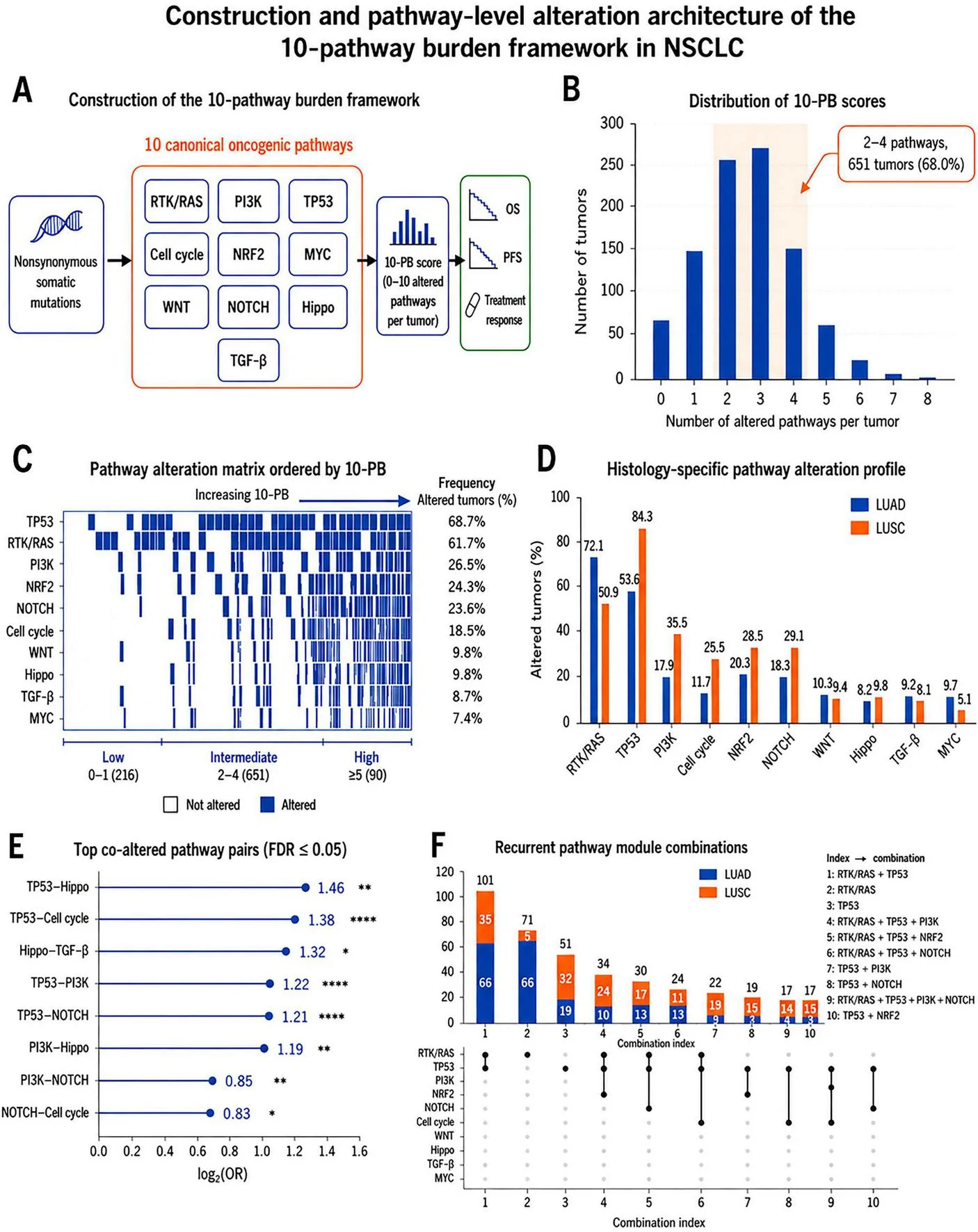
Construction and pathway-level mutational landscape of 10-PB. **(A)** Definition of 10-PB as the number of altered canonical oncogenic pathways per tumor. **(B)** Distribution of 10-PB scores in the discovery cohort. **(C)** Pathway alteration matrix ordered by increasing 10-PB. **(D)** Alteration frequencies and histology-specific pathway profiles. **(E,F)** Pairwise co-alteration and recurrent pathway-module analyses. *P < 0.05, **P < 0.01, ****P < 0.0001.

To complement gene-level mutation counts and TMB-based assessment, we established a 10-PB framework to quantify the breadth of mutation-based oncogenic pathway involvement in NSCLC. The study design, including the discovery cohort and external validation cohorts, is summarized in Figure 1. Nonsilent somatic mutations were mapped to 10 canonical oncogenic pathways, and the 10-PB score was defined as the number of altered pathways per tumor, ranging from 0 to 10 (Figure 2A).

In the discovery cohort, 10-PB scores varied across tumors, with most tumors carrying alterations in 2–4 pathways (Figure 2B). The pathway alteration matrix ordered by increasing 10-PB showed that dispersed gene-level mutations could be summarized into pathway-level patterns, with a broader range of altered pathways in tumors with higher 10-PB (Figure 2C). TP53 and RTK/RAS were the most frequently altered pathways, followed by PI3K, NRF2, NOTCH, and cell cycle. Histology-stratified analysis showed distinct pathway profiles between LUAD and LUSC. LUAD showed a more RTK/RAS-oriented pattern, whereas LUSC showed broader involvement of TP53, PI3K, cell cycle, NRF2, and NOTCH pathways (Figure 2D). Pairwise and recurrent module analyses identified co-altered pathway pairs and repeated pathway combinations, including RTK/RAS + TP53, RTK/RAS alone, TP53 alone, and RTK/RAS + TP53 + PI3K (Figures 2E,F). Representative gene-level analyses were consistent with these subtype-specific pathway profiles (Supplementary Figure 1). These results indicate that 10-PB provides a concise description of pathway-level mutation patterns in NSCLC.

### 3.2 Higher 10-PB was associated with poorer survival beyond TMB-based stratification

**FIGURE 3:**
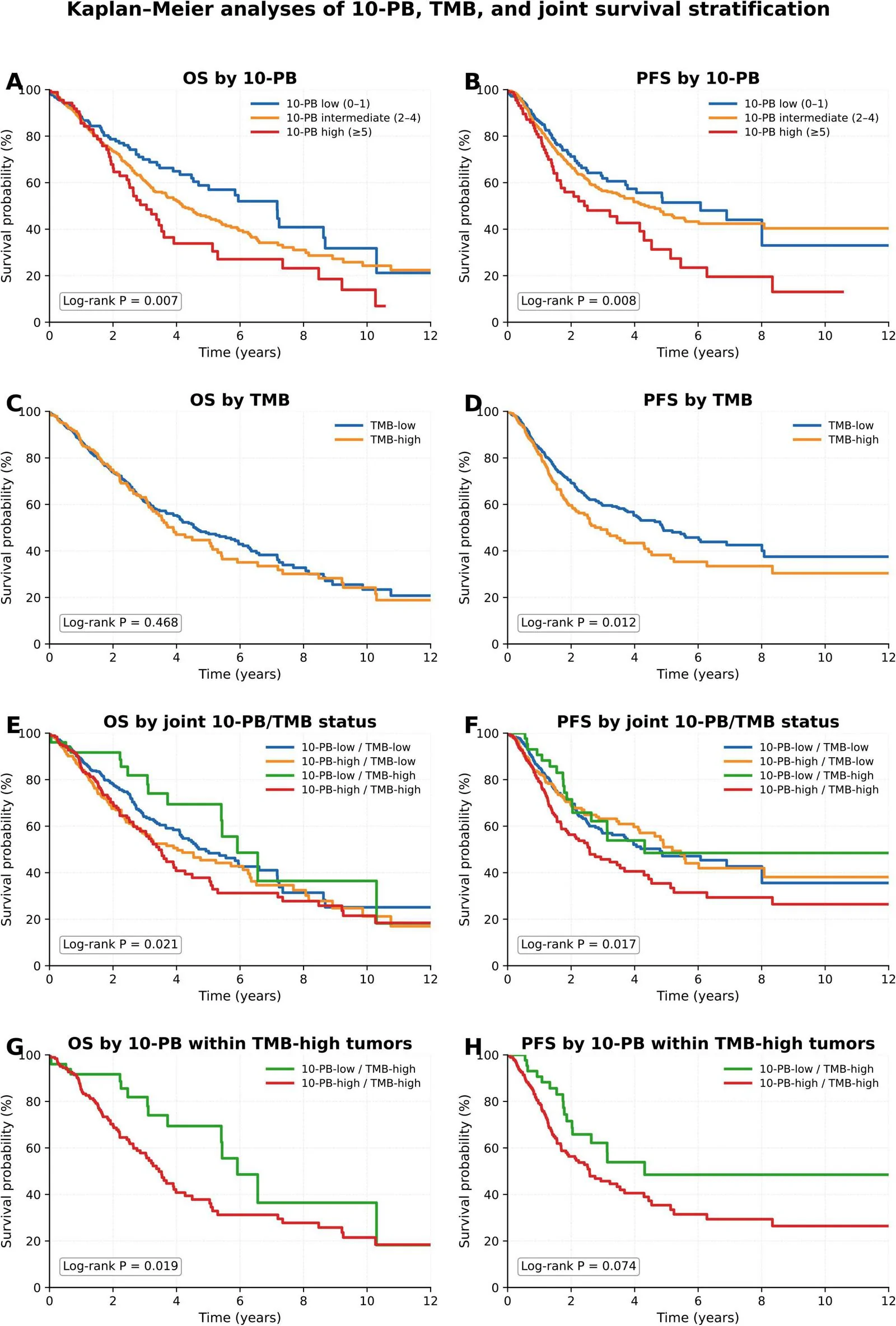
Survival stratification by 10-PB and TMB. **(A,B)** Kaplan–Meier analyses of OS and PFS according to 10-PB group. **(C,D)** Kaplan–Meier analyses of OS and PFS according to TMB group. **(E,F)** Joint stratification by 10-PB and TMB for OS and PFS. **(G,H)** Survival stratification by 10-PB within the TMB-high subgroup.

We next evaluated the survival relevance of 10-PB in the discovery cohort using Kaplan–Meier analyses. Patients with higher 10-PB had significantly shorter OS than those with lower pathway burden (log-rank *P* = 0.007; Figure 3A). A similar pattern was observed for PFS, where higher 10-PB was associated with poorer progression-free survival (log-rank *P* = 0.008; Figure 3B).

We then compared 10-PB-based stratification with TMB-based stratification. TMB did not significantly stratify OS in this cohort (log-rank *P* = 0.468; Figure 3C), although TMB-high tumors showed shorter PFS (log-rank *P* = 0.012; Figure 3D). Joint stratification by 10-PB and TMB separated patients into clinically distinct survival groups for both OS and PFS (Figures 3E,F). Within the TMB-high subgroup, 10-PB retained additional stratification for OS and showed a similar adverse trend for PFS (Figures 3G,H). These findings suggest that 10-PB and TMB reflect related but non-equivalent dimensions of tumor genomic complexity.

### 3.3 10 -PB provided prognostic information complementary to TMB for OS and PFS

**FIGURE 4:**
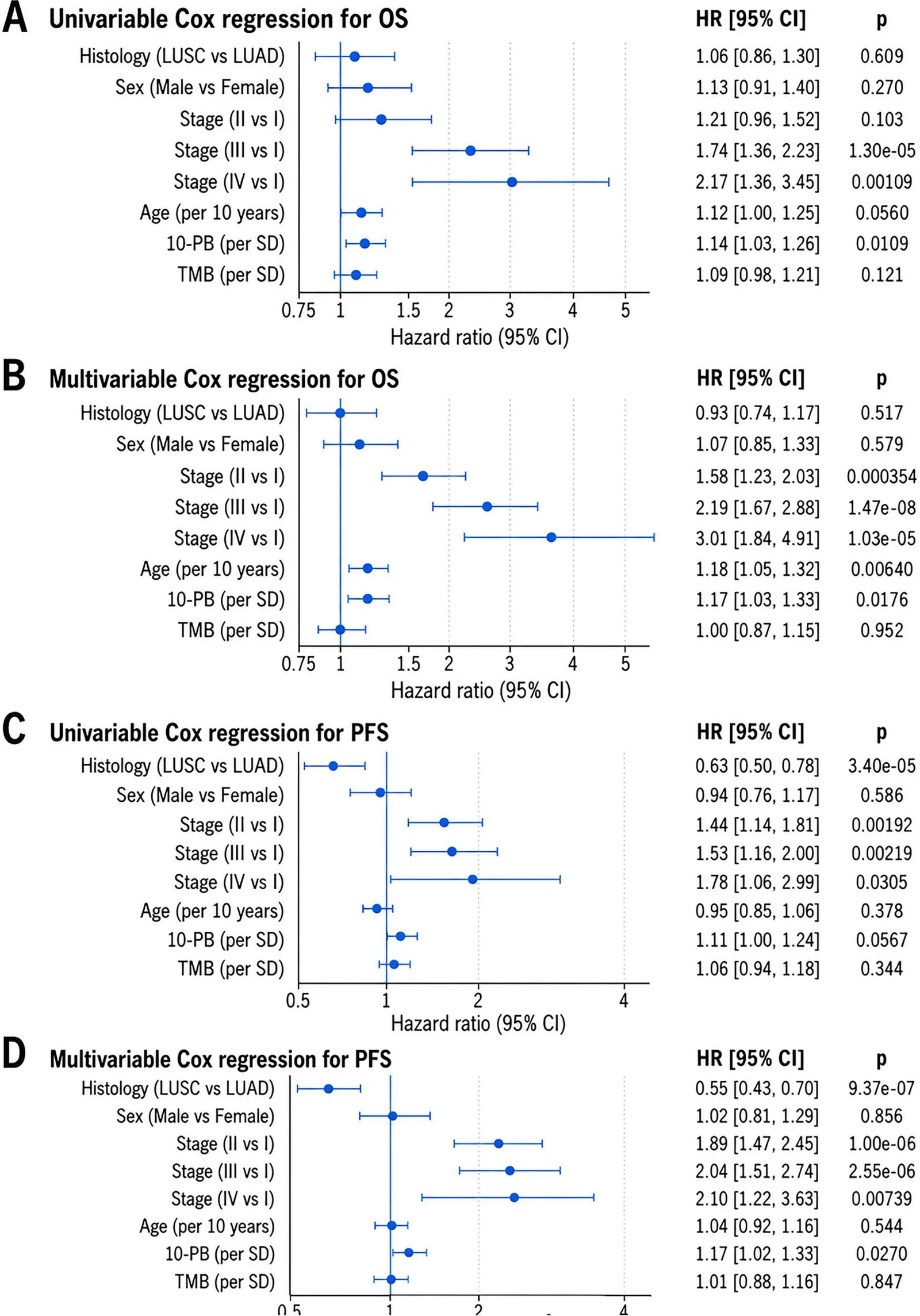
Tumor mutational burden (TMB)-independent prognostic value of 10-PB. **(A,C)** Univariable Cox regression analyses for OS and PFS. **(B,D)** Multivariable Cox regression analyses adjusted for histology, sex, stage, age, and TMB.

Cox regression analyses were performed to determine whether the survival association of 10-PB was independent of clinicopathologic factors and TMB. In univariable analysis, higher 10-PB was associated with poorer OS (HR per SD = 1.14, 95% CI: 1.03–1.26, *P* = 0.0109), whereas TMB was not significant (HR per SD = 1.09, 95% CI: 0.98–1.21, *P* = 0.121; Figure 4A). After adjustment for histology, sex, stage, age, and TMB, 10-PB remained independently associated with OS (HR per SD = 1.17, 95% CI: 1.03–1.33, *P* = 0.0176), while TMB showed no independent association (HR per SD = 1.00, 95% CI: 0.87–1.15, *P* = 0.952; Figure 4B).

For PFS, 10-PB showed a borderline association in univariable analysis (HR per SD = 1.11, 95% CI: 1.00–1.24, *P* = 0.0567; Figure 4C) and became significant after multivariable adjustment (HR per SD = 1.17, 95% CI: 1.02–1.33, *P* = 0.0270; Figure 4D). TMB was not independently associated with PFS in the adjusted model (HR per SD = 1.01, 95% CI: 0.88–1.16, *P* = 0.847). Together, the survival models indicate that 10-PB carries prognostic information beyond standard clinicopathologic variables and TMB.

### 3.4 10 -PB showed an exploratory association with adverse treatment response

**FIGURE 5:**
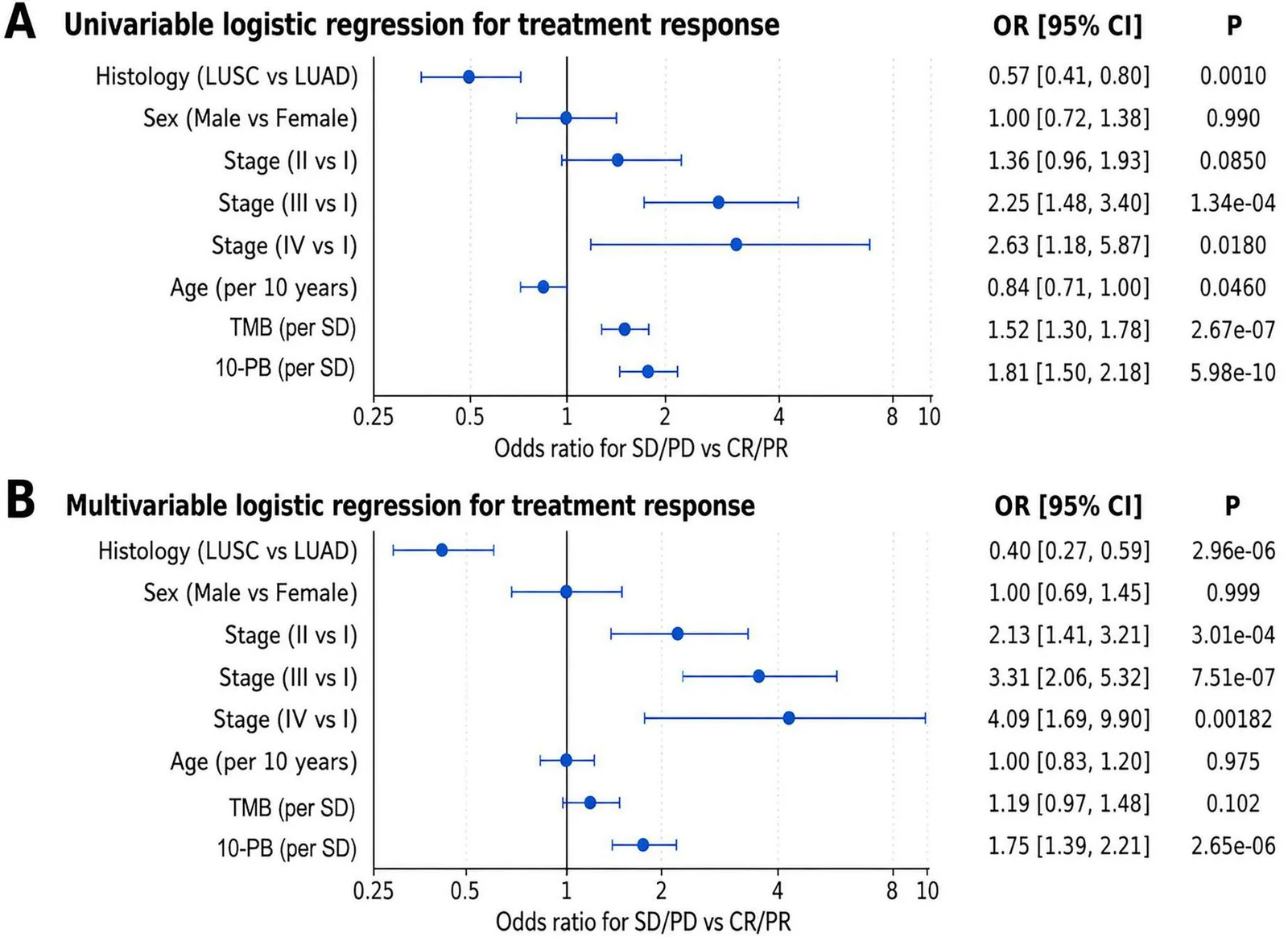
Association of 10-PB with adverse treatment response. **(A)** Univariable logistic regression analysis of adverse treatment response, defined as SD/PD versus CR/PR. **(B)** Multivariable logistic regression analysis adjusted for histology, sex, stage, age, and TMB.

We next assessed whether 10-PB was associated with treatment response. Adverse response was defined as SD/PD compared with CR/PR. In univariable logistic regression, higher 10-PB was associated with adverse response (OR per SD = 1.81, 95% CI: 1.50–2.18, *P* = 5.98 × 10-10; Figure 5A). TMB was also associated with adverse response in the univariable model (OR per SD = 1.52, 95% CI: 1.30–1.78, *P* = 2.67 × 10-7; Figure 5A).

After adjustment for histology, sex, stage, age, and TMB, 10-PB remained associated with adverse response (OR per SD = 1.75, 95% CI: 1.39–2.21, *P* = 2.65 × 10-6; Figure 5B). In contrast, the association between TMB and adverse response was attenuated and was no longer statistically significant after adjustment (OR per SD = 1.19, 95% CI: 0.97–1.48, *P* = 0.102; Figure 5B). Given the retrospective and potentially heterogeneous nature of the response annotations, this finding was interpreted as exploratory.

### 3.5 10 -PB provided incremental outcome-related information beyond TMB

**FIGURE 6:**
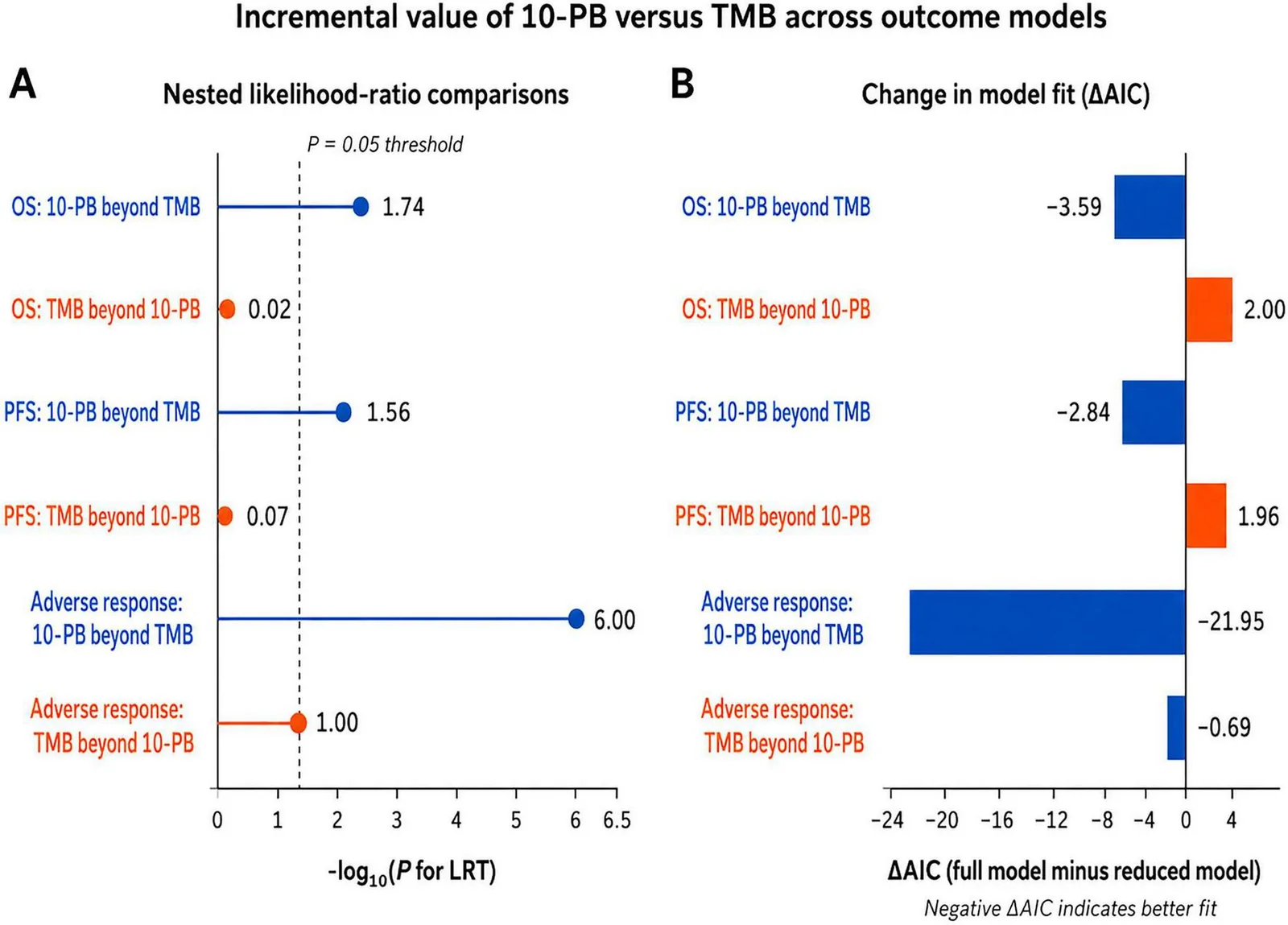
Incremental model value of 10-PB beyond TMB. **(A)** Nested likelihood-ratio tests comparing the added value of 10-PB and TMB across OS, PFS, and adverse response models. **(B)** Changes in Akaike information criterion after adding 10-PB or TMB.

We further compared the incremental value of 10-PB and TMB across outcome models using nested likelihood-ratio tests and changes in Akaike information criterion (ΔAIC). For OS, adding 10-PB to a model already containing TMB significantly improved model fit, whereas adding TMB to a model containing 10-PB provided little additional improvement (Figure 6A). A similar pattern was observed for PFS. Consistently, ΔAIC decreased after adding 10-PB beyond TMB for both OS and PFS, while adding TMB beyond 10-PB did not improve model fit (Figure 6B). In the adverse response model, 10-PB showed a larger likelihood-ratio signal and greater AIC reduction than TMB. These model comparisons indicate that pathway breadth and mutation load are not interchangeable measures.

### 3.6 Bootstrap and continuous trend analyses supported the robustness of the 10-PB survival signal

**FIGURE 7:**
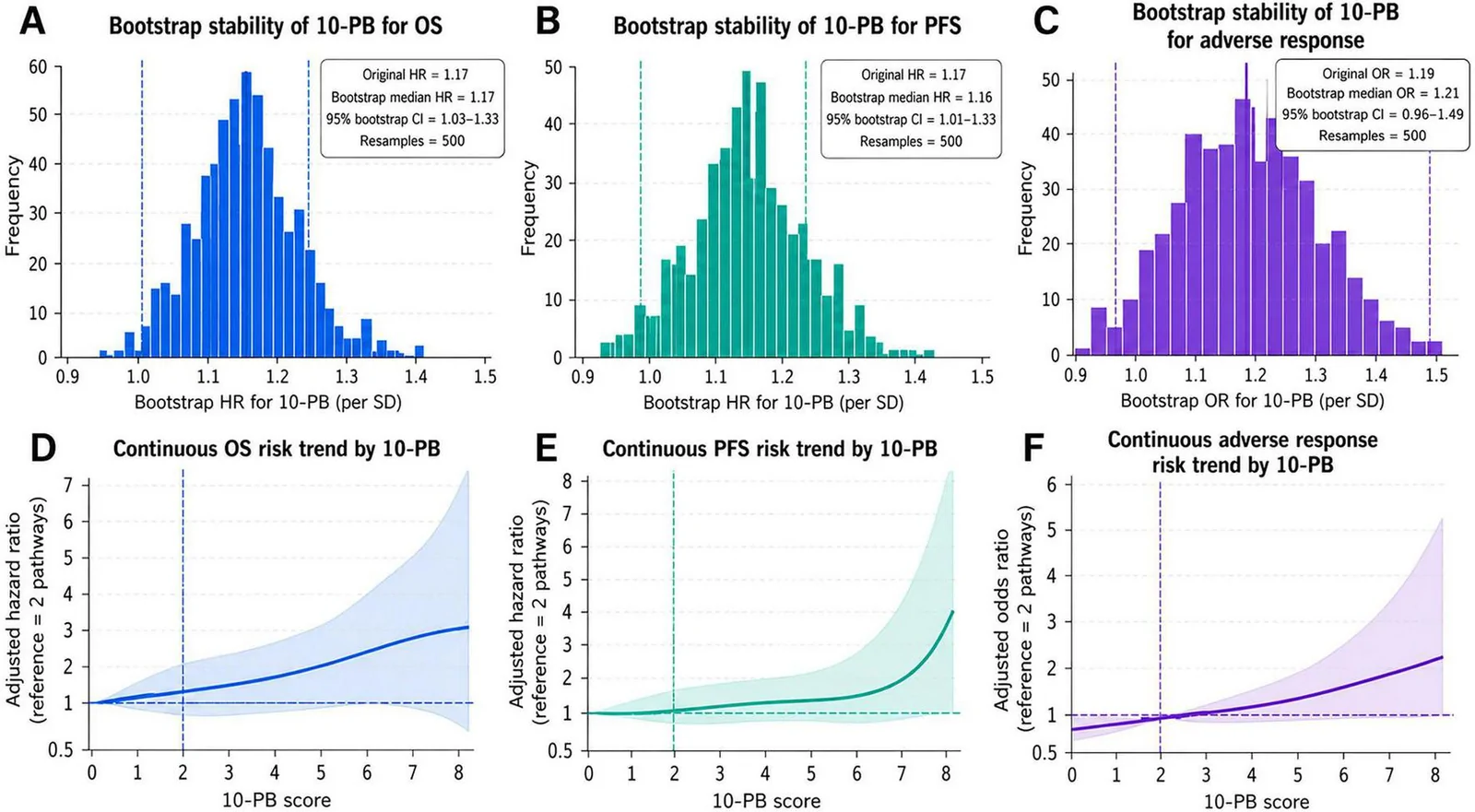
Bootstrap stability and continuous risk trends of 10-PB. **(A–C)** Bootstrap distributions of 10-PB effect estimates for OS, PFS, and adverse response. Solid lines indicate original estimates, and dashed lines indicate 95% bootstrap confidence intervals. **(D–F)** Continuous adjusted risk trends for OS, PFS, and adverse response, with 10-PB = 2 as the reference.

Bootstrap resampling and continuous-risk trend analyses were performed to evaluate the stability of the 10-PB associations. Across 500 resamples, the OS association remained stable, with an original HR of 1.17, a bootstrap median HR of 1.17, and a 95% bootstrap CI of 1.03–1.33 (Figure 7A). Similar stability was observed for PFS (original HR = 1.17; bootstrap median HR = 1.16; 95% bootstrap CI: 1.01–1.33; Figure 7B). The adverse-response analysis showed a positive but less stable trend because the bootstrap confidence interval crossed the null value (Figure 7C). Continuous-risk analyses showed increasing adjusted risk with higher 10-PB scores for OS and PFS, with a positive trend for adverse response (Figures 7D–F). These results support the OS and PFS findings, while the adverse-response result should remain exploratory.

### 3.7 Endpoint-specific pathway components showed model-based associations with survival and response endpoints

**FIGURE 8:**
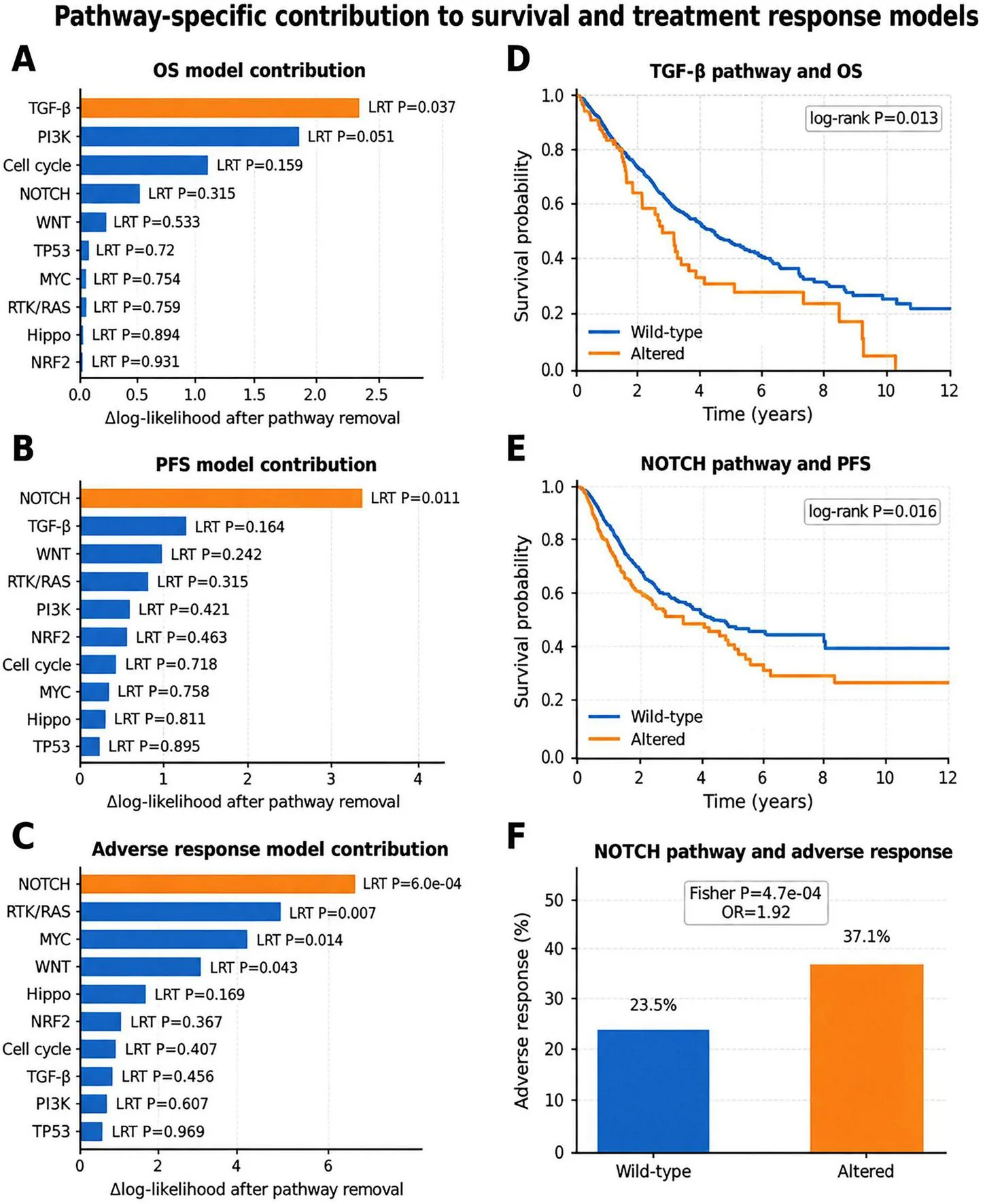
Endpoint-specific pathway associations with clinical outcomes. **(A–C)** Leave-one-pathway-out analyses for OS, PFS, and adverse response models. **(D)** OS according to TGF-β pathway alteration status. **(E)** PFS according to NOTCH pathway alteration status. **(F)** Adverse response according to NOTCH pathway alteration status.

We next examined whether the clinical associations of 10-PB were related uniformly to all pathways or were concentrated in specific pathway components. In the OS model, leave-one-pathway-out analysis showed that removing the TGF-β pathway produced the largest decrease in model fit (LRT *P* = 0.037), followed by PI3K (LRT *P* = 0.051; Figure 8A). Consistently, TGF-β pathway alterations were associated with shorter OS in Kaplan-Meier analysis (log-rank *P* = 0.013; Figure 8D).

For PFS, NOTCH showed the strongest model-based association (LRT *P* = 0.011; Figure 8B), and NOTCH-altered tumors had shorter PFS than NOTCH-wild-type tumors (log-rank *P* = 0.016; Figure 8E). In the adverse-response model, NOTCH again showed the largest model-based contribution (LRT *P* = 6.0 × 10-4), followed by RTK/RAS, MYC, and WNT (Figure 8C). NOTCH alterations were also associated with a higher adverse-response rate than wild-type tumors (37.1% vs. 23.5%; Fisher exact *P* = 4.7 × 10-4; OR = 1.92; Figure 8F). These analyses suggest endpoint-specific patterns, with TGF-β more closely associated with OS and NOTCH more closely associated with PFS and adverse response.

### 3.8 External validation supports the reproducibility of 10-PB and endpoint-specific pathway associations

**FIGURE 9:**
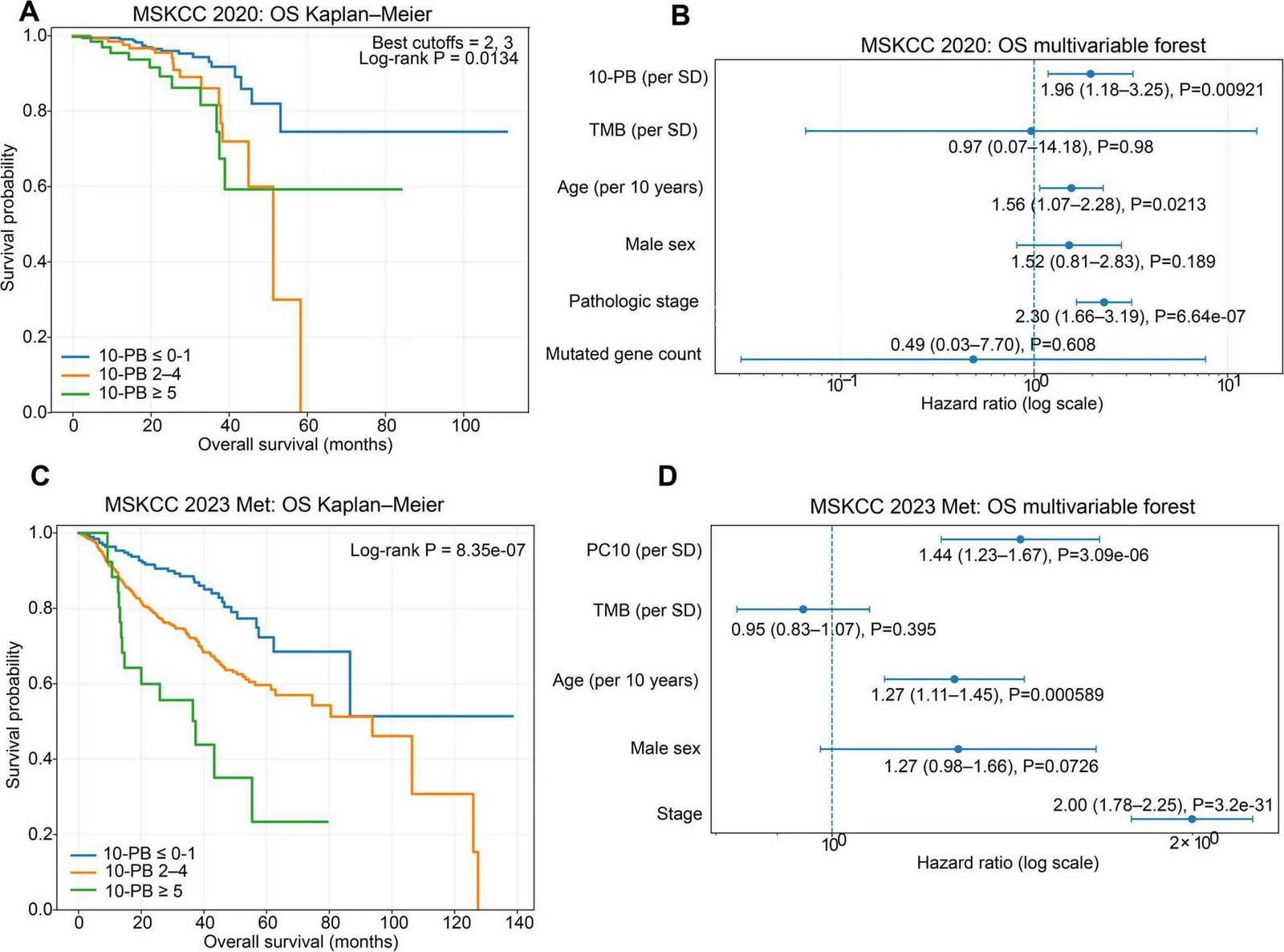
External validation of 10-PB for OS. **(A,B)** Kaplan–Meier and multivariable Cox analyses of OS in the MSKCC 2020 NSCLC cohort. **(C,D)** Kaplan-Meier and multivariable Cox analyses of OS in the MSKCC 2023 metastatic NSCLC cohort.

We first evaluated the prognostic reproducibility of 10-PB in independent NSCLC cohorts. In MSKCC 2020, higher 10-PB was associated with poorer OS in Kaplan-Meier analysis and remained independently associated with OS after multivariable adjustment (log-rank *P* = 0.0134; HR per SD = 1.96, 95% CI: 1.18–3.25, *P* = 0.00921; Figures 9A,B). Similar findings were observed in the MSKCC 2023 metastatic NSCLC cohort, where higher 10-PB was associated with poorer OS and remained significant in the adjusted Cox model (log-rank *P* = 8.35 × 10-7; HR per SD = 1.44, 95% CI: 1.23–1.67, *P* = 3.09 × 10-6; Figures 9C,D).

**FIGURE 10:**
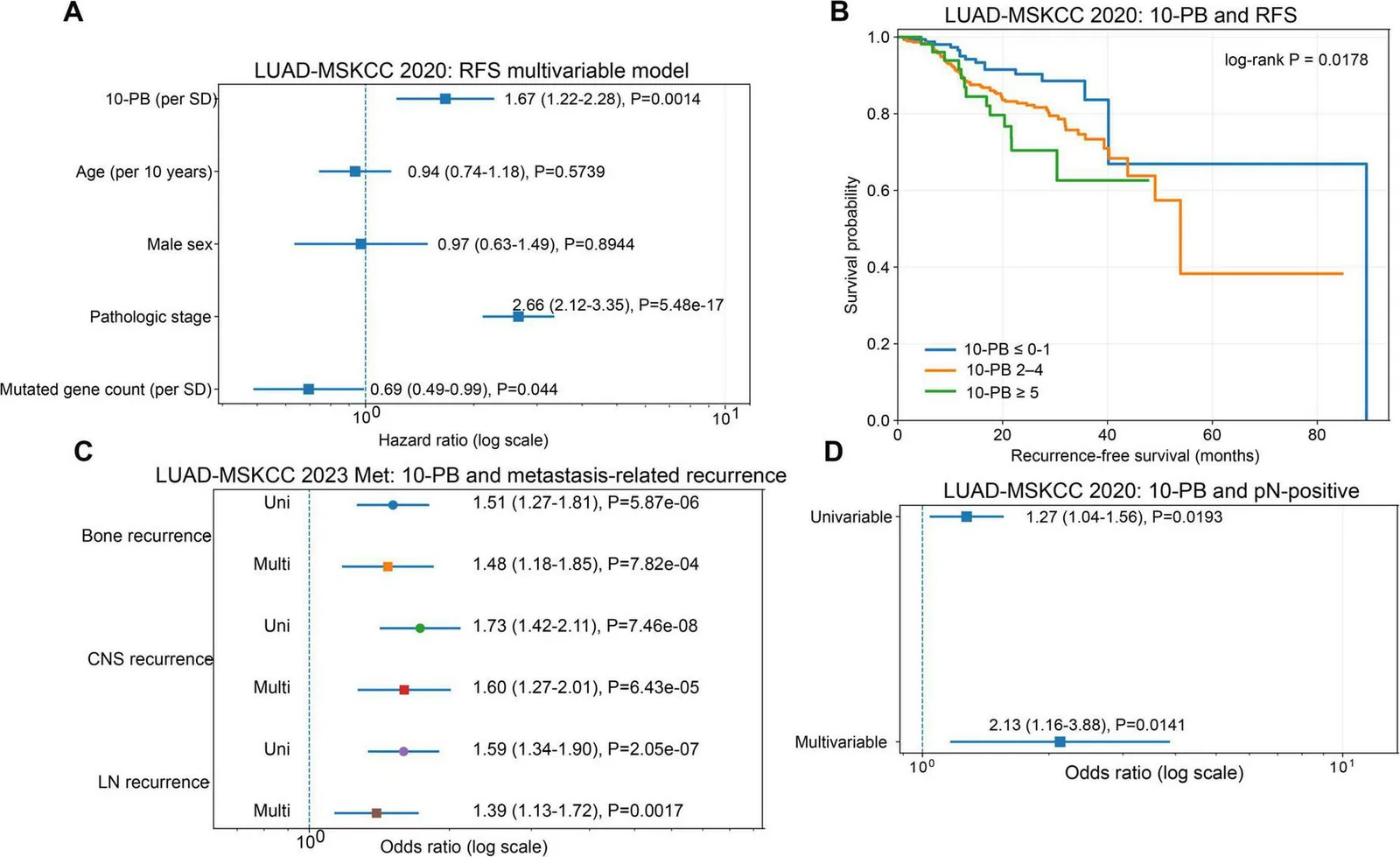
Validation of 10-PB in LUAD recurrence- and dissemination-related cohorts. **(A,B)** Multivariable Cox and Kaplan–Meier analyses of recurrence-free survival in the LUAD-MSKCC 2020 cohort. **(C)** Associations between 10-PB and bone, central nervous system, and lymph-node recurrence in the LUAD-MSKCC 2023 metastatic cohort. **(D)** Logistic regression analysis of pN-positive status in the LUAD-MSKCC 2020 cohort.

We then assessed 10-PB in LUAD-focused recurrence-related validation cohorts. In LUAD-MSKCC 2020, higher 10-PB was associated with shorter recurrence-free survival in both multivariable Cox and Kaplan–Meier analyses (HR per SD = 1.67, 95% CI: 1.22–2.28, *P* = 0.0014; log-rank *P* = 0.0178; Figures 10A,B). Higher 10-PB was also associated with bone, CNS, and lymph-node recurrence in LUAD-MSKCC 2023 metastatic cases, as well as pN-positive status in LUAD-MSKCC 2020 (Figures 10C,D).

**FIGURE 11:**
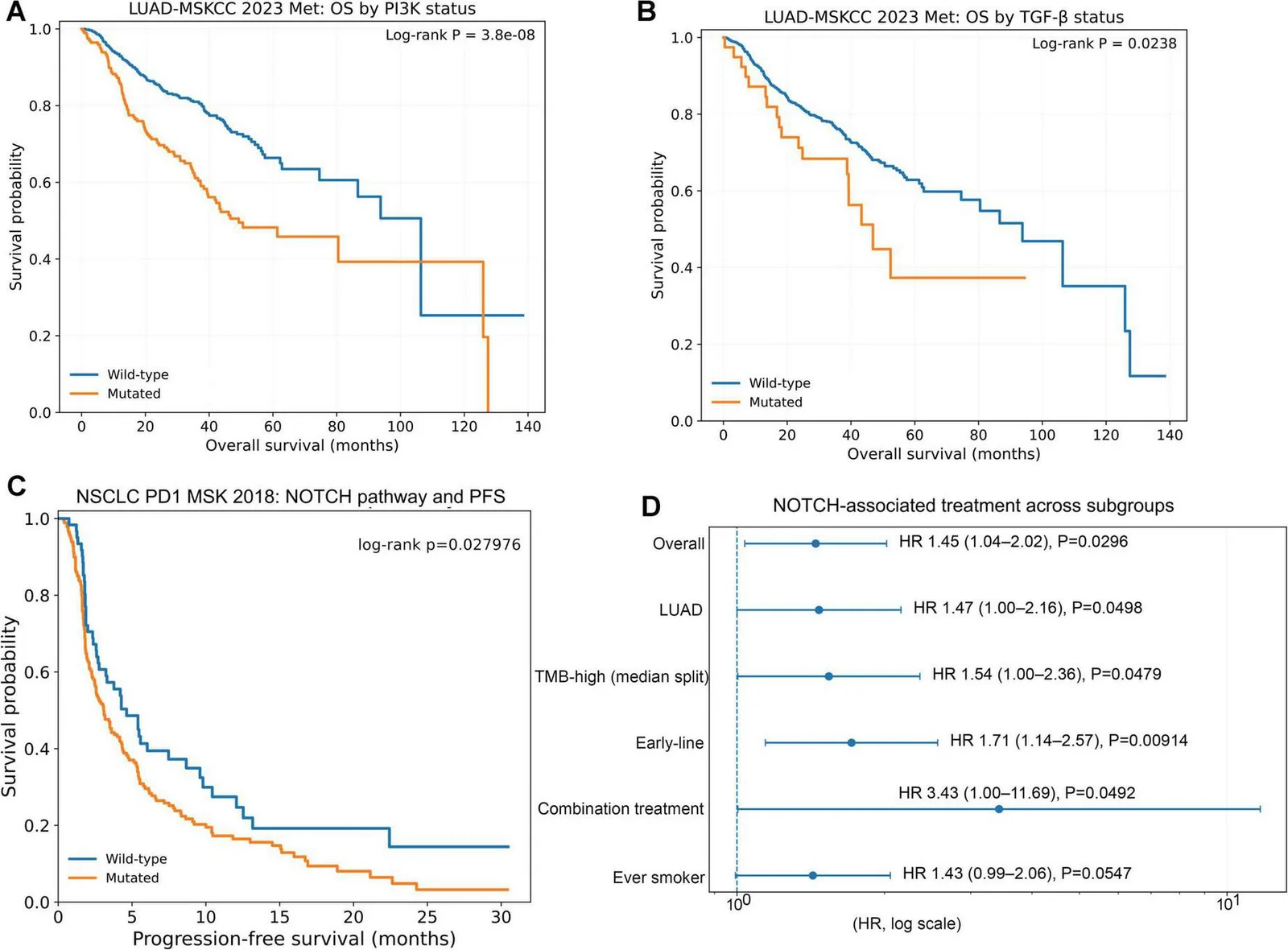
External validation of endpoint-specific pathway associations. **(A,B)** OS according to PI3K and TGF-β pathway alteration status in the LUAD-MSKCC 2023 metastatic cohort. **(C)** PFS according to NOTCH pathway alteration status in the NSCLC PD-1 MSK 2018 cohort. **(D)** Subgroup Cox analysis of NOTCH pathway alteration and PFS in the NSCLC PD-1 MSK 2018 cohort.

Finally, we examined whether selected endpoint-specific pathway findings were supported in external datasets. In LUAD-MSKCC 2023 metastatic cases, PI3K and TGF-β pathway alterations were associated with poorer OS (Figures 11A,B). In the NSCLC PD-1 MSK 2018 cohort, NOTCH pathway alteration was associated with shorter PFS (log-rank *P* = 0.027976; Figure 11C), with a consistent adverse direction across clinical subgroups (Figure 11D). These validation analyses were generally consistent with the discovery-cohort findings.

## 4 Discussion

This study used a 10-pathway framework to describe the somatic mutational architecture of NSCLC at the pathway level. Gene-level heterogeneity was summarized into recurrent pathway-level alteration patterns, with TP53 and RTK/RAS as the most frequently altered pathways and with distinct profiles in LUAD and LUSC. The cumulative breadth of mutation-based pathway involvement, measured by the 10-PB score, was associated with OS, PFS, and treatment response, and the survival associations were supported in independent validation cohorts. These results suggest that 10-PB captures a dimension of mutational complexity that is related to, but not identical with, single-gene alterations or overall mutation load. Endpoint-specific analyses further suggested that NOTCH was more closely associated with progression-related and response-related endpoints, whereas TGF-β and PI3K were more closely associated with OS in model-based analyses.

Non-small cell lung cancer mutational heterogeneity showed a nonrandom organization at the pathway level. Gene-level mutation profiles followed the expected long-tail pattern, with a small number of recurrently altered genes and many low-frequency events. Although informative, this structure is difficult to interpret clinically because rare alterations may converge on shared signaling processes. By aggregating nonsilent mutations into 10 canonical oncogenic pathways, the present analysis provided a pathway-level view of which programs were altered and how broadly they were involved within individual tumors.

The predominance of co-alteration over mutual exclusivity is consistent with the idea that several oncogenic programs may operate together during tumor development and progression (15–17). In this cohort, pathway co-involvement appeared more common than strict pathway substitution. This does not establish functional cooperation, but it suggests that pathway-level summaries may capture organization that is less apparent from single-gene mutation lists alone (18, 19).

The subtype-specific analyses were also informative. LUAD and LUSC differed not only in mutation burden but also in pathway configuration (7, 20). LUAD was enriched for RTK/RAS-related alterations and RTK/RAS-containing modules, largely driven by genes such as KRAS and EGFR. In contrast, LUSC showed broader involvement of TP53-, PI3K-, Cell cycle-, NRF2-, and NOTCH-related alterations, together with TP53-centered pathway modules (15, 21–23). These findings suggest that histologic differences in NSCLC are reflected not only by individual driver genes but also by how recurrent alterations are distributed across signaling programs. This interpretation remains inferential, but it is consistent with the view that LUAD and LUSC follow different pathway-level evolutionary patterns.

The value of 10-PB lies in its distinction from conventional mutation-count measures. Mutation burden and mutated-gene count describe how many events are present, whereas 10-PB describes how broadly those events involve canonical oncogenic pathways. Two tumors with comparable mutation loads may therefore have different 10-PB scores if their mutations are distributed across different numbers of pathways. In this study, higher 10-PB was associated with worse OS and PFS after adjustment for histology, sex, stage, age, and TMB. This association suggests that the clinical signal of 10-PB is not simply a reflection of a larger number of mutations. The graded Kaplan-Meier patterns and external validation results further support its potential use as a compact pathway-breadth measure.

The treatment-response analysis should be interpreted more cautiously. Higher 10-PB was associated with increased odds of SD/PD after covariate adjustment, but response annotations were retrospective and may reflect mixed treatment settings. Therefore, these data do not establish treatment prediction or causality. A possible explanation is that tumors with broader pathway involvement have more alternative signaling routes under therapeutic pressure, but this remains hypothesis-generating. In the pathway-specific analyses, NOTCH was more closely associated with PFS and adverse response, whereas PI3K and TGF-β were more closely associated with OS. These patterns suggest that the 10-PB signal may arise from several pathway components rather than from a single dominant pathway.

The endpoint-specific findings provide a useful way to interpret the 10-PB signal. PI3K and TGF-β showed stronger model-based associations with OS, which may relate to processes involved in long-term tumor aggressiveness, microenvironmental remodeling, invasion, or disease progression. NOTCH was more closely associated with PFS and treatment response, suggesting a possible relationship with early progression or adaptive resistance states (20, 24–26). RTK/RAS and MYC also appeared in the response model, consistent with a possible role of proliferative signaling and pathway reprogramming under treatment pressure (15). These interpretations remain exploratory because the analyses estimate statistical contributions within fitted models rather than direct biological mediation.

From a clinical perspective, 10-PB may complement established clinicopathologic and molecular variables rather than replace them (27). Stage remains central to prognosis, and individual driver alterations remain essential for therapeutic decision-making. 10-PB should not be considered a stand-alone biomarker at this stage. Its potential value is as a compact description of mutation-based pathway breadth when interpreted together with conventional clinical and genomic features (28, 29). Prospective validation, preferably with functional pathway-activity data, will be required before considering clinical application.

Several limitations should be considered. First, the 10-PB score gives equal weight to all altered pathways, although pathways differ in size, prevalence, and biological relevance. Second, because pathway calls were derived from somatic mutations, 10-PB reflects mutation-based pathway involvement rather than direct pathway activation, signaling output, or pathway dependency. Third, treatment-response analyses were based on retrospective annotations and heterogeneous therapeutic settings. Finally, leave-one-pathway-out analyses compare relative model contribution but do not establish mechanistic causality. Future studies incorporating copy-number, transcriptomic, proteomic, phosphoproteomic, and functional data may help determine whether mutation-based 10-PB corresponds to actual pathway activity.

## 5 Conclusion

This study characterized NSCLC mutational heterogeneity using a unified 10-pathway framework. By aggregating nonsilent mutations into canonical oncogenic pathways, we identified recurrent pathway-level alteration patterns with histology-specific features. The 10-PB score was associated with OS, PFS, and treatment response after adjustment for clinicopathologic variables and TMB, with supportive evidence from external cohorts. Compared with TMB, 10-PB describes the breadth of mutation-based oncogenic pathway involvement rather than mutation load alone. NOTCH was more closely associated with PFS and adverse response, whereas PI3K and TGF-β were more closely associated with OS in model-based analyses. These findings suggest that 10-PB may provide information complementary to TMB for NSCLC outcome stratification, pending further prospective and functional validation.

## Data Availability

The datasets analyzed in this study are publicly available from TCGA/GDC (https://portal.gdc.cancer.gov/) and cBioPortal (https://www.cbioportal.org/), including TCGA LUAD/LUSC and the external MSK cohorts used for validation.

## Supplementary material

Supplementary Figure 1 Gene-level context for pathway-level alteration calls. Representative mutated genes assigned to each canonical oncogenic pathway were compared between LUAD and LUSC to contextualize the pathway-level alteration calls.
